# Analgesic Efficacy of Pfannenstiel Incision Infiltration with Ropivacaine 7.5 mg/mL for Caesarean Section

**DOI:** 10.1155/2010/542375

**Published:** 2010-07-27

**Authors:** N. K. Nguyen, A. Landais, A. Barbaryan, M. A. M'Barek, Y. Benbaghdad, K. McGee, P. Lanba

**Affiliations:** ^1^Department of Anaesthesia and Intensive Care, Victor Dupouy Hospital, 95107 Argenteuil, France; ^2^Department of Obstetrics and Gynaecology, Victor Dupouy Hospital, 95107 Argenteuil, France

## Abstract

*Background*. Pain after Caesarean delivery is partly related to Pfannenstiel incision, which can be infiltrated with local anaesthetic solutions. *Methods*. A double- blind randomized control trial was designed to assess the analgesic efficacy of 7.5 mg/mL ropivacaine solution compared to control group, in two groups of one hundred and forty four parturients for each group, who underwent Caesarean section under spinal anaesthesia: group R (ropivacaine group) and group C (control group). All parturients also received spinal sufentanil (2.5 *μ*g). *Results*. Ropivacaine infiltration in the Pfannenstiel incision for Caesarean delivery before wound closure leads to a reduction of 30% in the overall consumption of analgesics (348 550 mg for group R versus 504 426 mg for group C with *P* < .05), especially opioids in the first 24 hours, but also significantly increases the time interval until the first request for an analgesic (4 h 20 min ± 2 h 26 for group R versus 2 h 42 ± 1 h 30 for group C). The *P* values for the two groups were: *P* < .0001 for paracetamol, *P* < .0001 for ketoprofen and *P* for nalbuphine which was the most significant. There is no significant difference in the threshold of VAS in the two series. *Conclusion*. This technique can contribute towards a programme of early rehabilitation in sectioned mothers, with earlier discharge from the post-labour suite.

## 1. Introduction

The number of Caesarean sections is increasing progressively in European countries with the aim of improving fetal prognosis; about 20% of deliveries are performed under Caesarean section [1, 2]. Caesarean section commonly induces moderate to severe pain lasting 48 hours [3–5]. Opioids are commonly used for relief of postoperative pain after Caesarean section, either by intrathecal administration prior to section or parenteral administration postoperatively. 

The addition of opioids intrathecally prolongs the effects of the spinal anaesthesia and reduces the dose of local anaesthetic required, thus reducing the hemodynamic effects which are deleterious to the fetus. However, to obtain analgesia of good quality and of long duration then higher doses of opioids have to be used. But the risk of complications such as respiratory depression, urinary retention, pruritus, nausea, and vomiting can preclude patient's comfort [6–9]. 

On the other hand, nonopioid systemic analgesics are not powerful enough to allow effective pain control after Caesarean section [10, 11]. Other techniques of postoperative analgesia, such as epidural morphine or local anaesthetics also have limits because they require prolonged clinical surveillance. 

 The aim of this study is to examine the quality of analgesia provided by the infiltration of a solution of 7.5 mg/mL ropivacaine in Pfannenstiel incision for Caesarean sections by analysing the overall analgesic consumption of both opioid and nonopioid agents, in the first 24 postoperative hours, in order to show whether ropivacaine infiltration leads to a reduction in the need for postoperative analgesic medication.

## 2. Methods

A total of two hundred and eighty eight ASA 1 parturients, who underwent elective Caesarean section under spinal anaesthesia, were included in this prospective double-blind randomised control study. Parturients were excluded from the study if they had an emergency procedure or if the procedure was performed under general or epidural anaesthesia, or if they had a contraindication to the administration of nonsteroidal anti-inflammatory drugs (NSAID's).

 The study received the approval of the ethical committee and parturients were included after written informed consent. Parturients were assigned randomly into two groups: group R (ropivacaine group) and group C (control group). Randomisation was performed using random number allocation and sealed envelopes, and each group consisted of one hundred and forty four parturients. 

 Spinal anaesthesia was performed at L3-L4 with 8–10 mg of a hyperbaric bupivacaine 5 mg/mL solution, according to the height of the patient (8 mg for patients measuring from 1 m 50 to 1 m 59, 9 mg for patients from 1 m 60 to 1 m 69, and 10 mg for patients taller than 1 m 70). The administration of sufentanil 2.5 *μ*g was standardized for all the patients. 

 A urinary catheter was inserted systematically before the Caesarean section and was left in place for 24 hours.

 All Caesarean sections were performed using a Pfannenstiel incision, with peritoneal opening. Before skin closure, infiltration with 30 mL of ropivacaine 7.5 mg/mL (225 mg) was performed in group R: 10 mL for the aponeurosis and 10 mL subcutaneously on each of the upper and lower edges of the incision, using a 30 mL syringe and a 23 G subcutaneous needle. This infiltration in three distinct parts was chosen to standardize the practice and reduce operator-related differences, since six surgeons participated in the operations. Group C did not receive any infiltration before skin closure.

 In both groups further postoperative analgesia was administered in the recovery room or in the post-labour suite when pain score, evaluated systematically every 2 hours and at every analgesic request by the patient, was ≥4 on the VAS. 

 Initially, paracetamol 1 g IV was administered when the patients complained of pain and then repeated every 6 hours over 24 hours. Intravenous ketoprofen at a dose of 3 mg/kg was administered secondarily every eight to twelve hours, when the patients requested further pain relief and if the VAS ≥4, and when paracetamol administration alone was ineffective. If this regimen was insufficient, nalbuphine 15–20 mg was given intravenously over one hour every 6 hours.

## 3. Statistics

From our previous experience (and data in the literature) we calculated that a number of one hundred and forty four patients in each arm was necessary, assuming a significant difference of 30% or more in systemic analgesic consumption, with a type I error of 0.05 and a power of 0.9. Data were analyzed using the Statistical Package for the Social Science Version (SPSS for Windows, release 10.0; SPSS, Chicago, IL). A Kolmogorov-Smirnov test was used, and stratified distribution plots were examined to verify the normality of distribution of continuous variables. Baseline characteristics (age, gender, duration of surgery, duration of anaesthesia, ASA scores, weight, height, and BMI) and the delay before rescue analgesic were compared across treatment groups using 2-way analysis of variance or the Fischer's exact test. The number of patients receiving rescue analgesics at a fixed interval was analyzed by a 2-way ANOVA and *post hoc* comparisons at various times using Bonferroni's type I error correction for multiple comparisons. Data were expressed as mean ± SD. The level of significance was set at *P* < .05. No adaptive interim analysis was performed during the study.

## 4. Results

Parturients in the two groups were comparable for demographics and VAS scores (Table 1). All patients enrolled completed the study.

**Figure 1:**
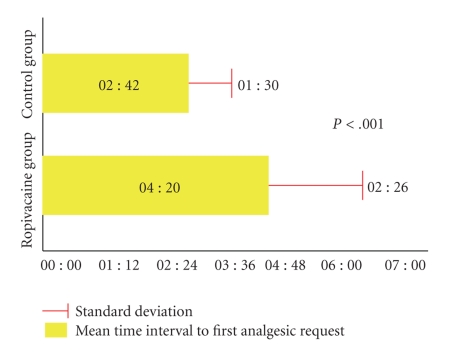
Interval before the first analgesic administration (Pain free interval).

 The comparative analysis was carried out between the two groups for each type of analgesic administered, taking as a reference point the moment when the spinal anaesthesia was performed, the request for analgesics and VAS scores by blocks of two hours, the overall consumption for each type of analgesic, and for all three types of analgesics overall (Tables 2 and 3 and Figure 1).

**Figure 2:**
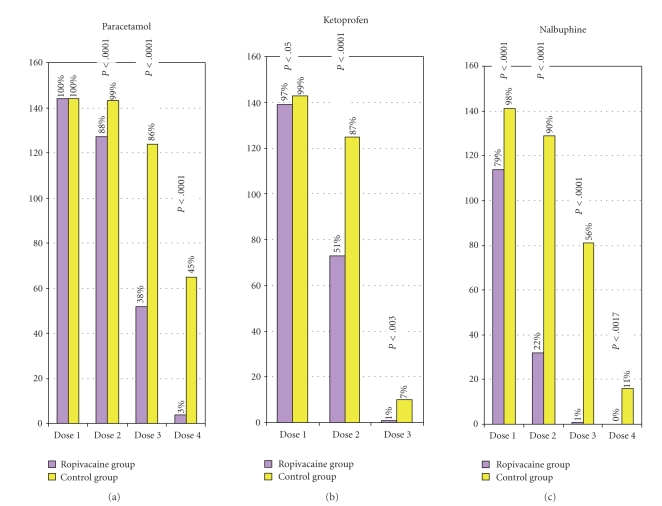
Percentage of patients needing analgesic administration over 24 hours.

**Figure 3:**
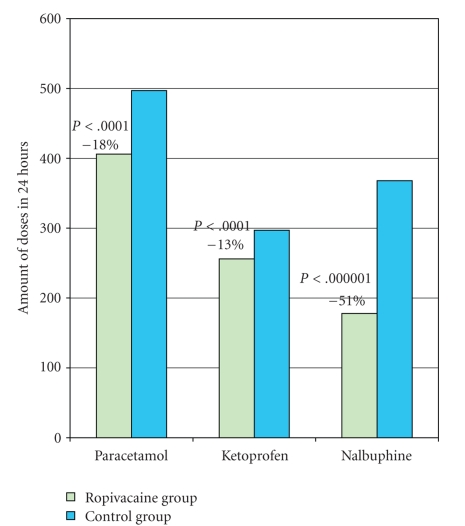
Postoperative analgesic consumption.

 The percentage of parturients who requested the first dose of intravenous paracetamol is identical in the two groups. The percentage of patients who were given paracetamol was significantly less on the second and the third time of administration in the ropivacaine group. The percentage of patients who received ketoprofen was also lower in the ropivacaine group at the second and the third time of administration as well as the percentage of patients who requested nalbuphine at the corresponding intervals (Figures 2 and 3).

In both groups, the nurse in charge of each patient monitored the ventilation, the presence of nausea and vomiting, of paralytic ileus and pruritis during the first 24 postoperative hours and recorded her findings on a chart.

 Spontaneous ventilation monitored by a pulse oximeter for the first 6 postoperative hours detected no sign of postoperative respiratory depression. No case of nausea or vomiting was reported in the first 24 hours, either during fasting or after reintroduction of food, 8 hours after surgery, which was well tolerated by all the patients. No sign of abdominal distension or of absence of intestinal transit was recorded by the nursing staff. No case of pruritis was reported by the patients.

The urinary catheter was removed at the 24th postoperative hour and normal bladder function resumed with no case of urinary retention noted among the 288 patients in our study.

## 5. Discussion

 This study documents that surgical incision infiltration with ropivacaine 7.5 mg/mL significantly prolongs by 2 hours and 26 minutes the pain-free interval after Caesarean section and decreases the rescue analgesic demand by 30% (Figures 1, 2 and 3). 

 Previous studies have demonstrated that local anaesthetic infiltration was effective after parietal surgery such as inguinal hernia repair [12–16].

 In 1998 Moiniche et al. published a series of five studies concerning incisional local anaesthesia for postoperative pain relief after abdominal operations [17]. All these studies showed that pain scores were reduced with VAS decreased by around 25–50 mm.

 In three studies, the pain scores were reduced after surgery, from 1 to a maximum 4 hours. In the study carried out by Sinclair et al., the pain scores were reduced in the first 24 hours, but not from the 24th to 48th hour [18]. In the study by Tverskoy et al., pain scores were reduced up until 48th hour after surgery [19]. In the four studies in which the time interval from infiltration to the first request for analgesics was evaluated, the duration of analgesia was significantly prolonged at 2–7 hours. Thereafter, Tverskoy et al. used a fixed analgesic regimen in their study design, but in the four other studies a significant reduction (approximately 50%) in supplementary analgesic consumption was found compared to the control group.

 Other studies have previously demonstrated that incisional infiltration was effective after Caesarean section [20, 21]. In addition the technique is safe since several studies have demonstrated that the plasma concentration of ropivacaine remains below the toxic threshold provided the dose is limited to less than 300 mg [15, 22].

 One may argue that spinal sufentanil and/or morphine may ensure effective analgesia without the need for an alternative technique such as abdominal wall infiltration. However, the hydrosolubility of morphine leads to its prolonged presence in the cerebrospinal fluid with effects lasting from 18 to 24 hours. It slowly penetrates the spinal cord and the dorsal horn [23, 24] thus there is a risk of delayed respiratory depression because of cephalic spread as well as other secondary effects such as nausea, vomiting, and pruritis [6, 7, 25]. Spinal opioids not only produce side effects, but in the dose range that is acceptable, cannot guarantee 24 hour analgesia [26, 27]. The infiltration of local anaesthetics at the surgical incision allows us to use a lower dose of intrathecal morphine and so limit its side effects. For this reason we used sufentanil at the dose of 2.5 *μ*g, despite its shorter duration of action compared with either morphine or fentanil. 

 From the 1980s morphine has been used by epidural injection after Caesarean section in doses from 2 to 8 mg [28, 29]. However epidural administration of morphine frequently leads to pruritis, nausea, and vomiting, even with doses <1,25 mg [28–30]. Respiratory depression is the complication which has the greatest risk, its frequency being proportional to the dose administered. In the study by Writer et al. in 1985, respiratory depression was observed in 7% of patients who received 5 mg of morphine by the epidural route, which imposes prolonged monitoring of ventilation up to 24 hours after the injection [31]. Some hospitals cannot provide such surveillance, and so the use of this technique is limited. 

 The technique of PCEA with low dose of ropivacaine limits patient mobility because of the encumbrance of the machines used and so is not a technique of choice in our practice. 

 The TAP block is not within the capability of all anaesthesiologists, as it requires initiation and experience in the technique. Its use is also limited because of the risk of intraperitoneal injection or lesions of intraabdominal organs, as described by Farooq and Carey [32], and Jankovic et al. [33]. Echo-guided TAP block gives more security but requires the availability of an echograph in the obstetrical operating room.

 Since the beginning of the 90s, many studies have been carried out on the quality of postoperative analgesia obtained with continuous infusions, instillations and, more recently, infiltrations of ropivacaine in surgical wounds [34–36]. These new techniques have led to a better quality of analgesia and a significant decrease in the consumption of systemic analgesics in the first 24 postoperative hours. 

 Incisional infiltration also has a limited duration of action (less than 5 hours) but it contributes to the decrease in demand for systemic analgesics thereafter. Nevertheless, this result suggests that a continuous parietal infiltration could be adapted to extend the duration of analgesia to the whole painful postoperative period. The use of this analgesic technique does not lead to any increase in wound dehiscence or infection.

On the other hand, the continuous administration of local anaesthetic into the surgical wound can be more restrictive because the dressings need to be changed more frequently, because of leakage of the anaesthetic solution from the wound.

 Numerous studies have shown the analgesic efficacity of ropivacaine infiltration into the surgical wound without overdose, cardiovascular, or neurological toxicity [36–40]. However, the peak blood concentration is proportional to the dose injected and the concentration of the solution used, as has shown Wulf et al. in 1999 in his study on ilio-inguinal nerve block using different concentration of ropivacaine: 0.2%, 0.5%, or 0.75% in hernia repair surgery [22].

 As far as ropivacaine toxicity is concerned, the epileptogenic threshold in man is unknown. In healthy volunteers, after intravenous infusion of ropivacaine, neurological signs appear with concentrations of 4 300 ng ± 600 ng/mL (3 400–5 300 ng/mL). The peak is obtained after 13–15 minutes of infusion, while it is obtained after 20 minutes for epidural analgesia and 21(± 9) minutes after intercostal nerve block.

 From among all the techniques of postoperative analgesia presented here, the infiltration of ropivacaine into the Pfannenstiel incision represents the technique best adapted to our practice, because of its indisputable efficacy and the simplicity and ease of realisation by all our obstetricians. It favorises early mobility in the patients, from the 6th–8th postoperative hour, thus allowing the sectioned mothers to visit their babies sooner in the case of premature births where the neonates are often in intensive care units. The mother-child contact can thus be established earlier and this contributes to their psychologic well-being. 

 Early mobility leads to more rapid intestinal activity and so, to more rapid oral intake of liquids and solids. In our hospital all patients who have undergone elective Caesarean section and who have no bleeding or digestive complications are fed 8 hours after surgery.

## 6. Conclusion

Postoperative infiltration of the surgical incision in Caesarean section with ropivacaine 7.5 mg/mL gives effective analgesia for several hours and decreases systemic analgesic consumption. This technique could be considered as an integral part of the analgesic protocol in patients scheduled for Caesarean section. It aims to give optimal pain relief with minimal side effects, without interfering with the mother-child relationship, allowing breast feeding and favorising postoperative rehabilitation.

 Our study has shown no surgical postoperative complication related to this technique.

 Since this study, the protocol has been applied by all members of the obstetrical team for all Caesarean sections performed under spinal or general anaesthesia, except in cases of contraindications to the use of local anaesthetics.

## Figures and Tables

**Table 1:** Parturient's demographics and VAS scores evaluated every 2 hours.

	age (year)	height (cm)	weight before pregnancy (kg)	BMI	VAS
Ropivacaine group	31.7 ± 5.5	159.7 ± 6.8	66.6 ± 14.1	25 ± 6	5.2 ± 1.6
Control group	32.25 ± 5.4	161.4 ± 6.2	67.0 ± 14.9	26 ± 5	5 ± 1.4
*P* =	.22	.06	.41	.12	.10
Non significant	NS	NS	NS	NS	NS

**Table 2:** Statistical Analysis (1).

		Ropivacaine Group				Control Group			
Type of Analgesic		Number of patients	Analgesics over 24 h (mg)	Mean VAS	Standard deviation	Number of patients	Analgesics over 24 h (mg)	Mean VAS	Standard deviation
	Dose 1	144	144 000	5.2	2 : 26	144	144 000	5	2 : 42
Paracetamol	Dose 2	127	127 000	5.6	2 : 17	143	143 000	5.6	2 : 55
	Dose 3	52	52 000	5.5	5 : 06	124	124 000	5.5	4 : 09
	Dose 4	4	4 000	4	1 : 34	65	65 000	5.4	2 : 36

TOTAL		X	327 000	5.0	2 : 50	X	476 000	5.4	3 : 05

	Dose 1	139	13 900	5.7	3 : 58	143	14 300	5.8	3 : 22
Ketoprofen	Dose 2	73	7 300	5.6	0 : 26	125	12 500	5.9	3 : 49
	Dose 3	1	100	5	0 : 00	10	1 000	5.5	6 : 12

TOTAL		X	21 300	5	1 : 48	X	27 800	5.7	4 : 27

	Dose 1	114	180	6.2	4 : 34	141	250	6	17 : 26
Nalbuphine	Dose 2	32	50	6.1	4 : 18	129	220	6.1	5 : 48
	Dose 3	1	20	7	0 : 00	81	132	5.7	5 : 41
	Dose 4	0	0	0	0 : 00	16	24	5	11 : 22

TOTAL		X	250	6	4 : 26	X	626	5.7	10 : 04

TOTAL OVERALL		144	348 550	5.6	3 : 01	144	504 426	5.6	5 : 52

**Table 3:** Statistical analysis (2).

	Ropivacaine Group	Control Group	*P*
Mean VAS	5.2	5	NS
Mean time interval to first analgesic request (hours)	04 : 20	02 : 42	<.001
Standard deviation	02 : 26	01 : 30	
Analgesic consumption (mg)	348 550	504 426	<.001
